# A multi-view representation technique based on principal component analysis for enhanced short text clustering

**DOI:** 10.1371/journal.pone.0309206

**Published:** 2024-08-23

**Authors:** Majid Hameed Ahmed, Sabrina Tiun, Nazlia Omar, Nor Samsiah Sani

**Affiliations:** 1 CAIT, Faculty of Information Science and Technology, Universiti Kebangsaan Malaysia, Bangi, Selangor, Malaysia; 2 Ministry of Higher Education and Scientific Research, Baghdad, Iraq; Hebei University of Technology, CHINA

## Abstract

Clustering texts together is an essential task in data mining and information retrieval, whose aim is to group unlabeled texts into meaningful clusters that facilitate extracting and understanding useful information from large volumes of textual data. However, clustering short texts (STC) is complex because they typically contain sparse, ambiguous, noisy, and lacking information. One of the challenges for STC is finding a proper representation for short text documents to generate cohesive clusters. However, typically, STC considers only a single-view representation to do clustering. The single-view representation is inefficient for representing text due to its inability to represent different aspects of the target text. In this paper, we propose the most suitable multi-view representation (MVR) (by finding the best combination of different single-view representations) to enhance STC. Our work will explore different types of MVR based on different sets of single-view representation combinations. The combination of the single-view representations is done by a fixed length concatenation via Principal Component analysis (PCA) technique. Three standard datasets (Twitter, Google News, and StackOverflow) are used to evaluate the performances of various sets of MVRs on STC. Based on experimental results, the best combination of single-view representation as an effective for STC was the 5-views MVR (a combination of BERT, GPT, TF-IDF, FastText, and GloVe). Based on that, we can conclude that MVR improves the performance of STC; however, the design for MVR requires selective single-view representations.

## Introduction

Text data growth has attracted the interest of numerous researchers who want to discover effective methods of obtaining useful hidden information. Most of these data texts come in the form of short texts and need a special analysis compared with formally written ones [1, 2]. Short texts are typically concise, ranging from a few words to one or two sentences, often not exceeding a paragraph. They are structured differently and do not always have complete syntactic and contextual content compared to longer texts. The maximum length of short text may change according to the platform or the situation in which the text is utilized. In recent years, short texts such as tweets, social media posts, captions, and text messages have become increasingly popular among users to interact with friends, seek information, review items to obtain information, and exchange suggestions and opinions [3, 4]. The result is massive, unstructured text created daily on the web [5, 6]. Accordingly, the massive amount of these short texts on the web emphasises the importance of data mining techniques for extracting valuable information [7, 8]. Extracting knowledge from these texts is challenging due to the absence of contextual information and their chaotic nature, containing slang, emojis, misspellings, abbreviations, and grammatical errors. Accordingly, researchers have developed creative and efficient methods to address these challenges. One of these methods is text clustering, specifically short text clustering (STC).

STC is a data-mining method for short texts because it automatically identifies useful patterns in a large and disordered collection of texts [9]. Clustering methods seek to discover similarity patterns in corpus data by grouping similar texts together and organising them into logical and semantic structures [10, 11]. Therefore, STC has received considerable attention in recent years to address the main challenges of current STC techniques. These challenges include limited length, sparsity, and lack of information, which affect the representation of the text to be grouped [12–17]. However, STC is more complex than traditional text clustering. This complexity is primarily due to the sparseness of text representations in the original lexical space, which is exacerbated for short texts [1, 18]. The key to a successful STC is to develop an efficient approach for representing short texts suitable for clustering.

Text representation is a vital aspect of STC. Earlier research on STC commonly uses single-view representation techniques [19–21]; however, single-view representation on STC is unable to perform efficiently due to its failure to capture semantic similarities effectively [22]. This aspect is crucial to understanding the semantic links between words. Usually, in the previously presented methods, textual data views are represented using a single feature. This mechanism cannot capture all aspects of the text. Consequently, valuable information can be lost through different representations (views) [23]. The views correspond to different sources of the same document or parts of the document. To this end, considering various aspects of the data can improve the clustering quality [24]. Thus, the drawbacks of conventional text representation techniques can be overcome by multi-view representation (MVR). By taking into account multiple view representations (each view representation is associated with a feature space), also known as multi-view clustering, more and a variety of information can be captured, thus enhancing STC.

MVR uses different text representations to capture the semantic aspect and contextual meaning of the text data to overcome the lack of information in the short text, thus improving clustering algorithms. Multi-view clustering utilises many characteristic data views obtained from the MVR as an input. This mechanism combines many feature views to provide an optimal and more effective clustering model than conventional single-view clustering [25, 26]. Nevertheless, the challenge of taking MVR as the input for STC is to determine which combination of single views is optimal for STC.

Nevertheless, the currently available methods only take a single representation of all views based on the frequency of terms. This situation results in losing essential information and failing to capture text semantics [23]. Therefore, MVR is a promising approach for addressing the challenges facing STC. Where the MVR mechanism combines different representations or views by capitalising on their supplementary qualities [26]. Therefore, we can create a more robust representation of each short text piece by leveraging multiple sources of information, such as word embeddings, syntax features, and sentiment scores. This phenomenon results in more accurate and meaningful clusters, which inform business decisions, research findings, and more.

Herein, we propose a suitable combination of MVR for STC. In our work, different types of view representations (based on the types of data extraction) will be combined in different sets of combinations and learned using the same type of clustering methods. Then, the best consistent performance will be used as the evaluation method for identifying the best MVR. Experiments are conducted on three real-life datasets for analysis and validation of the performance of the proposed method. In this paper, the main contributions are as follows:

1. First, we build MVRs based on different single-view sets using fixed-length concatenation using PCA.
2. Second, we propose the most suitable MVR for STC by analyzing different sets of MVRs based on the performances of STC. Despite being a simple approach, our experiments demonstrate the effectiveness of MVRs with different datasets.

The remainder of this paper is organised as follows: Related work on multi-view clustering will be presented in Section 2. The design of combining single-view for MVR based on PCA will be described in Section 3. Then, the implementation of various types of MVR for STC will be explained in Section 4. Afterwards, the evaluation results and discussion are carried out in Section 5. We conclude our work and discuss future work in Section 6.

## Related studies

Recently, researchers have focused on short text representation, which is the most effective part of STC. Most models typically focus on representations of local word co-occurrences [15]. Several strategies, such as corpus-based measures [27] and knowledge-based metrics [28, 29], have been proposed to overcome sparsity and the lack of information challenges, but they come with limitations such as dependency on external resources, scalability issues, and the potential for biased or incomplete representations caused by context absence [30]. Another strategy focuses on improving textual data by using metadata or external data [28, 31]. However, these models can address sparsity issues, but they have drawbacks because they rely heavily on external information. Considering the representation problems associated with short text, new representation techniques suitable for STC must be developed. This study focuses on the STC based on the representation of short text. We summarized the related work into two main categories.

### Traditional representation in STC

In the field of STC, various traditional representation methods have been explored to overcome the challenges posed by the sparsity and ambiguity of short texts. [32] addressed the issue of clustering web documents by using semantically similar terms and a wiki-based k-nearest neighbour method, enhancing web search result clustering (WSRC). This approach was validated on standard datasets and demonstrated effectiveness with traditional clustering methods like k-means and k-medoids. [33] highlighted the difficulty in clustering and labeling microblog messages due to their short nature, proposing a framework that uses semantic knowledge bases like Wikipedia and Wordnet to enhance text representation and clustering. [5] introduced a BDM (Context-Aware Weighted Biterm) method for text distance measurement, utilizing corpus-wide word co-occurrence information to enrich document-level features and addressing the issue of sparse and ambiguous information in short texts. Lastly, [34] presented a corpus-based approach for STC that enriches text by leveraging conjugate definitions and a virtual generative procedure, adding new words with virtual term frequencies to short text documents. This method showed comparable performance to techniques relying on external information sources, underlining the potential of internal corpus-based enhancements in STC. These studies highlighted various methods to tackle the inherent challenges of STC. Still, they are constrained by dependencies on external knowledge sources, potential biases, challenges in handling context-specific nuances, and limitations in domain applicability.

### Deep learning representation in STC

In the field of STC, deep learning approaches have been explored to enhance text representation. [29] developed STC2, a self-learning neural network framework, to address incomplete text representation by incorporating semantic features and convolutional neural networks to process embedded words. The framework utilised an adaptive, unsupervised approach to integrate raw text attributes into the algorithm. The embedded words were processed through convolutional neural networks to capture deep feature representations. Nevertheless, further research is required to investigate the optimal selection and integration of semantic features within the proposed framework. Similarly, [35] introduced Contractive Autoencoders (CtAE) and a deep embedding clustering framework (DECCA) to enhance document clustering. CtAE focused on preserving relevant information and enhancing cluster locality, while DECCA aimed to capture the local manifold structure of input data. Despite their innovations, these methods face drawbacks, such as the complexity of selecting and integrating appropriate semantic features in STC2 and the challenge in DECCA to balance the preservation of relevant information with the need for effective data augmentation, posing potential risks of overfitting or losing critical nuances in the clustering process.

### Ensemble clustering methods based on MVRs

In the field of multi-view text clustering, recent advancements have focused on ensemble methods for integrating diverse text representations. [26] developed an ensemble method employing models like Latent Dirichlet Allocation (LDA), Vector Space Model (VSM), and Skip-gram to create multiple views, then applied the K-means algorithm for clustering. Combining cluster-based similarity partitioning with pairwise dissimilarity ensemble techniques, their method yielded improved clustering quality over single-view methods. However, the study did not thoroughly address the selection of views or evaluate the relevance and reliability of each view, leaving a gap in understanding the impact of view selection on clustering quality. Similarly, [36] introduced a method for merging clustering partitions using Kolmogorov complexity and an information theory model. This approach aimed for a complete merge of clusters, implementing a novel strategy for the order of merging. The method showed resilience to noise, integrating unsupervised ensemble learning with multi-view clustering. Yet, it could face challenges in determining the optimal merging strategy, particularly in handling complex datasets with poorly defined cluster boundaries.

The multi-view text clustering approach developed by [37] uses diverse text representations derived from advanced models like BERT to significantly enhance clustering quality, addressing the limitations of traditional methods that rely on term frequencies and miss deeper semantic relationships. This method employs multiple “views” of the data, each highlighting unique text aspects, and integrates them using a late fusion strategy to produce a coherent clustering outcome. However, one drawback is the integration strategy after independently clustering each view, which can lead to inadequate synthesis of different view representations. [38] proposed the MCoCo (Multi-level Consistency Collaborative Multi-View Clustering) method that uses feature space cluster assignments and semantic space alignments across views to guide the clustering process. However, this method has some problems. Achieving this alignment is challenging, primarily when the views are derived from heterogeneous sources or semantic labels are not well-defined or consistent across views. Also, this non-fusion method limits the ability to exploit the synergies between complementary views fully. In the work of [39] the Feature Concatenation Multi-view Subspace Clustering (FCMSC) method introduces multi-view clustering by leveraging the concept of feature concatenation to amalgamate multiple views into a unified representation. The method effectively deals with sample- and cluster-specific errors by including the *l*_2,1_ − norm in the objective function. However, the effectiveness of the method may be limited in scenarios where corruption is not sparse, as it relies on the assumption that such corruption can be addressed through the *l*_2,1_ − norm.

In the first and second categories of the presented methods, textual data views are represented using a single feature. This mechanism cannot capture all aspects of the text. Consequently, valuable information can be lost through different representations (views). The views correspond to different sources of the same document or parts of the document. To this end, considering various aspects of the data can improve the clustering quality [24]. The third category combines multiple clustering and obtains single consensus clustering. [40] showed that multi-view K-means have better experimental results than their single-view equivalents. The other views may make up for outliers and data noise in one view. Numerous studies have exploited the aspect of ensemble clustering data by incorporating multiple individual clustering algorithms to improve the clustering results [41, 42]. However, combining multiple clusters does not integrate the different representations. In addition, the literature has not addressed integrating various views of the same text document before the clustering phase. Therefore, considering different text representations as views can improve the clustering performance for the same document.

## The MVR based on PCA

**Fig 1:**
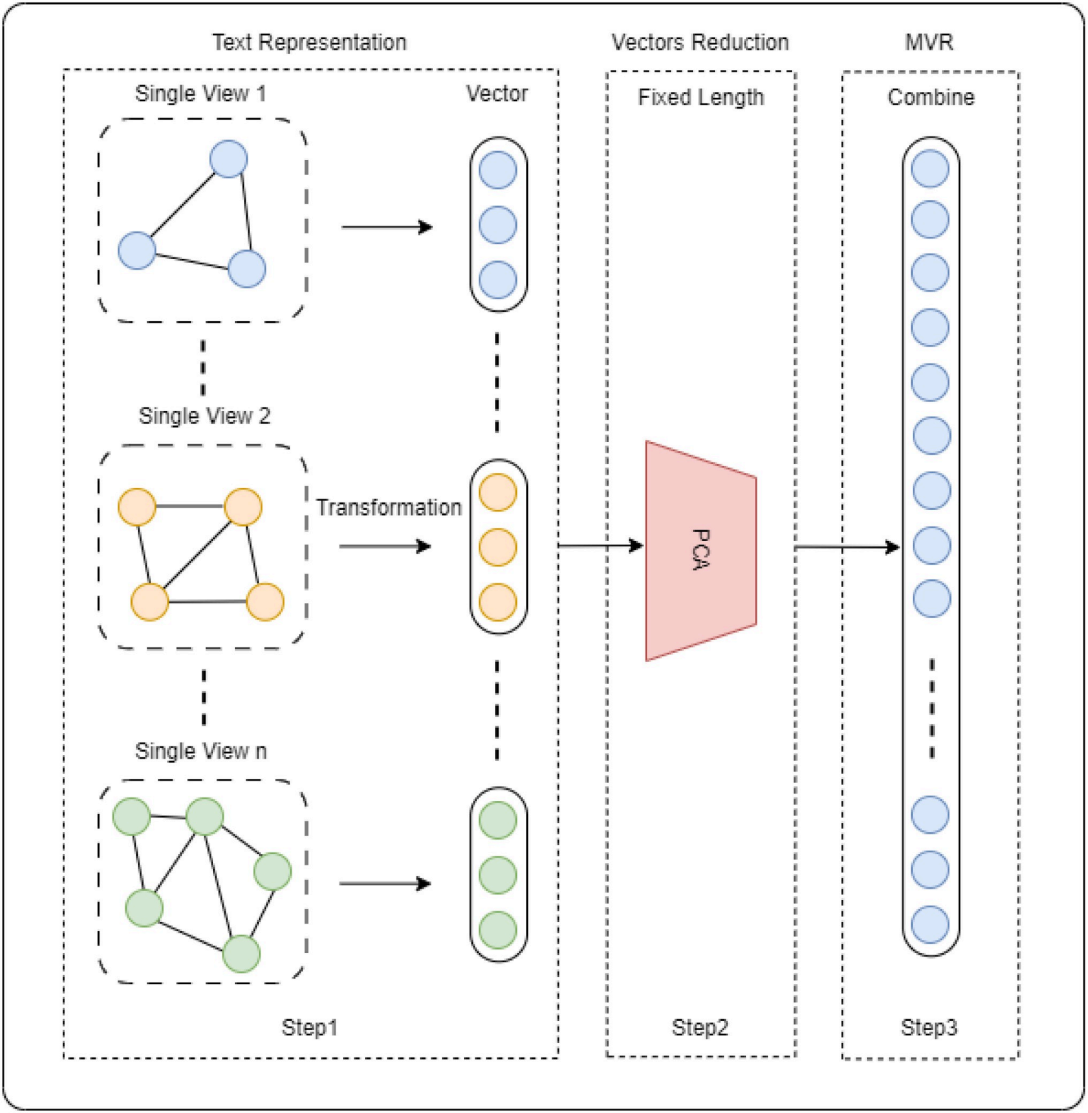
Proposed method to build MVR.

Our proposed MVR approach for STC aims to address the limitations of conventional approaches by capitalising on the complementary benefits of the various text representations. Meanwhile, approaches that solely rely on a single representation, such as TF-IDF or word embeddings, may overlook certain aspects of the text or struggle to manage short documents. For example, TF-IDF models are based predominantly on term frequencies and inverse document frequencies, which may not adequately capture complex semantic relationships between words. Although word embeddings, such as BERT and GPT, excel at capturing contextual information and semantic relatedness, they may not adequately address the challenges unique to short texts, such as a limited vocabulary or ambiguity. The MVR approach overcomes these limitations by combining multiple representations. Each representation provides distinctive strengths and perspectives, and their integration allows for a more complete and robust comprehension of short texts. Therefore, the MVR proposed for STC will improve the quality of clustering results. We designed our MVR by considering merging single-view representations from different kinds of ‘views’ of short text. Then, different kinds of ‘views’ are reduced into a fixed-length unit using PCA before being combined using a concatenation approach. Our MVR proposed approach consists of three steps, as shown in Fig 1.

### Step 1: Text representation (single view)

**Fig 2:**
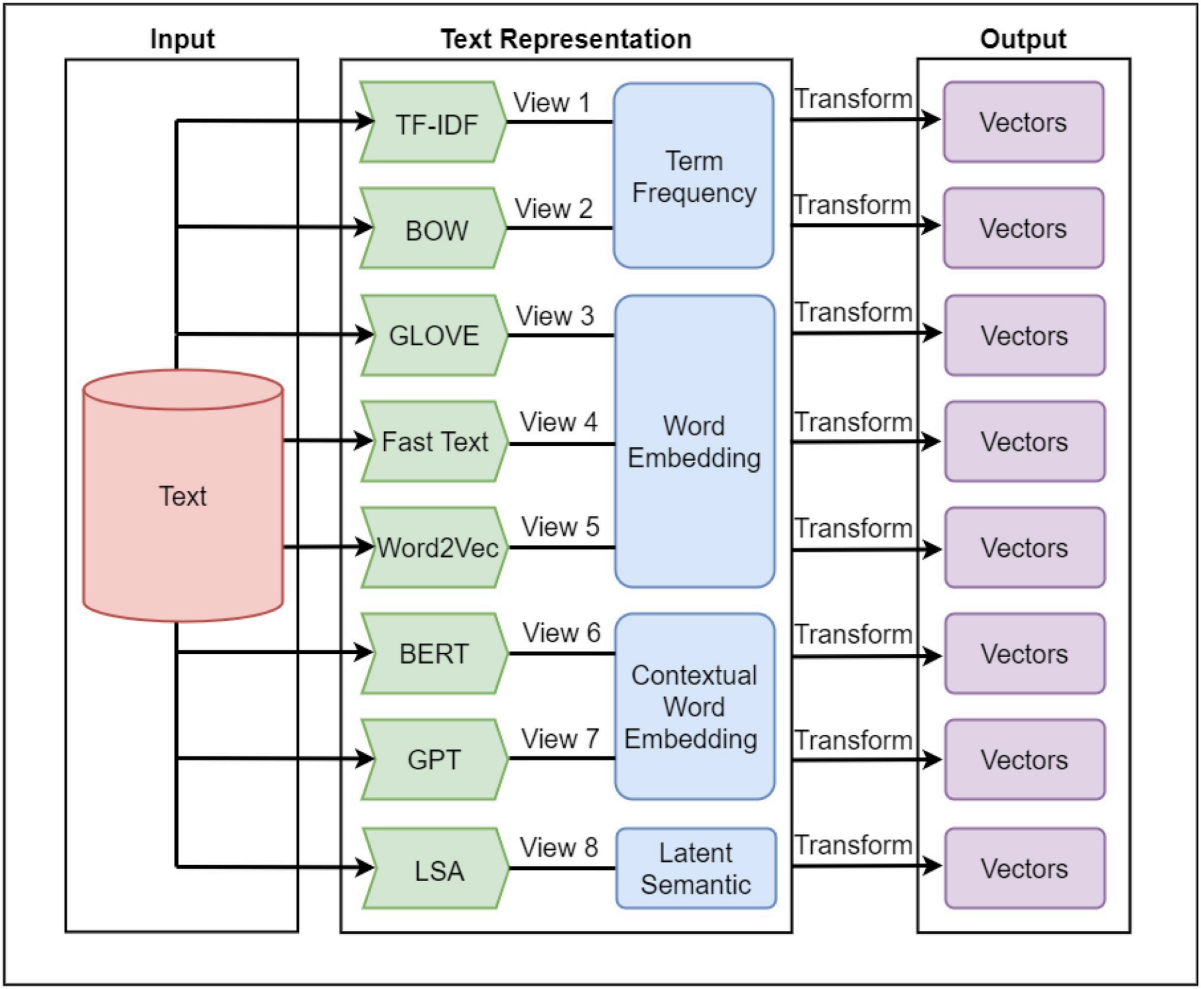
The selection of text representation based on the four types of views.

At this step, single-view representations that will be used for MVR are selected, and the selection must consider that the single-view has different types of ‘views’ on a short text. This is due to different ‘views’ (representations) capturing different aspects (hidden or unhidden information), which eventually aid in overcoming the issue of the lack of information in STC. In our work, we have chosen eight types of representations to capture four types of properties of a short text. The eight types of ‘views’ are based on four aspects of a short text, which are (i) the statistical property of a text (‘Term Frequency’), (ii) semantic property (‘Word Embedding’), (iii) contextual semantic property (‘Contextual Word Embedding’), and (iv) Latent Semantic (see Fig 2):

For the statistical property (‘**Term Frequency**’), we have selected the Term Frequency–Inverse Document Frequency (TF-IDF) and Bag of Words (BOW) representations as the ‘views’. TF-IDF is a text representation that highlights the importance of words in a document. TF-IDF assesses word significance based on frequency within a particular document and rarity across a dataset that assigns higher weights to unique and informative words. This dual consideration of term frequency (TF) and inverse document frequency (IDF) makes TF-IDF a valuable tool for distinguishing essential words in short texts for clustering within a corpus. The following are the equations (Eqs 1–3) that are used to transform the short text into TF-IDF representation [43].

$$\begin{array}{c} \text{TF}\left(t,d\right)=\frac{\text{Number}\;\text{of}\;\text{times}\;\text{term}t\text{appears}\;\text{in}\;\text{document}d}{\text{Total}\;\text{number}\;\text{of}\;\text{term}\;\text{in}\;\text{document}d} \end{array}$$ (1)

$$\begin{array}{c} \text{IDF}\left(t,D\right)=\text{log}(\frac{\text{Total}\;\text{number}\;\text{of}\;\text{documents}\;\text{in}\;\text{corpus}D}{\text{Number}\;\text{of}\;\text{documents}\;\text{with}\;\text{term}t}) \end{array}$$ (2)

$$\begin{array}{c} \text{TF-IDF}(t,d,D)=\text{TF}(t,d)\times \text{IDF}(t,D) \end{array}$$ (3)

As for Bag of Words (BOW) is a simple yet effective natural language processing and information retrieval model. In this model, a text is represented as an unordered set of words, where frequency counts are recorded, but grammar and word order are disregarded while maintaining diversity. This model is beneficial for tasks such as document classification and clustering [44]. Let *x*_*i*_ represent the frequency of each word *w* in the document *D* as shown in the following equation (Eq 4):

$$\begin{array}{c} x_{i}=\text{freg}\left(w_{i},D\right) \end{array}$$ (4)

Where frequency (*w*_*i*_, *D*) is the number of times the word *w*_*i*_ appears in the document *D*. For a binary representation, it would be:

$$\begin{array}{c} x_{i}=\{\begin{array}{cc} 1 & \text{if}\;w_{i}\;\text{appears}\;\text{in}\;D\\ 0 & \text{otherwise} \end{array} \end{array}$$ (5)

For semantic property (‘**Word Embedding**’), two types of views are selected: (i) Global Vectors for Word Representation (GloVe), (ii) FastText, and (iii) Word2Vec. GloVe is an unsupervised learning method for word vector representations, focusing on word co-occurrences across a large corpus. GloVe analyses global word pair frequencies, capturing broad usage patterns, and specific semantic relationships. GloVe creates word vectors through a co-occurrence matrix, optimizing these vectors to reflect the significance of words based on their joint occurrences in the corpus. Using GloVe, word relations can be represented regardless of what length and can be captured efficiently [45]. The following equation (Eq 6) is the formula to transform short text into GloVe representation. Where *V* is the size of the vocabulary, *w*_*i*_ and ${\tilde{w}}_{j}$ are the word and context word vectors respectively, *b*_*i*_ and ${\tilde{b}}_{j}$ are scalar biases for each word and context word, and *f*(*X*_*ij*_) is a weighting function.

$$\begin{array}{c} J=\sum_{i,j=1}^{V}\;f\left(X_{ij}\right){\left(w_{i}^{T}{\tilde{w}}_{j}+b_{i}+{\tilde{b}}_{j}-\text{log}X_{ij}\right)}^{2} \end{array}$$ (6)

As for FastText, it is a text representation based on a deep learning technique that converts short text into vectors representing semantic attributes. FastText operates by representing words as Skip-gram or sub-word units, predicting the likelihood of a word appearing in a given context. FastText maps each word in a document to a dense vector, capturing semantic features, and is particularly adept at handling out-of-vocabulary words, misspellings, and abbreviations through sub-word information [46]. Thus, FastText is effective for processing short texts with uncommon words or errors, as it can discern semantic attributes even from rare or misspelt words by summing the vectors of a word’s skip-gram components. The vector for a word *w* is obtained by summing the vectors of its constituent skip-gram. Where *G*_*w*_ is the set of skip-gram for the word *w*, and **Z**_*g*_ is the vector for the skip-gram *g* (see Eq 7).

$$\begin{array}{c} v_{w}=\sum_{g\in G_{w}}\;Z_{g} \end{array}$$ (7)

As for Word2Vec is a popular deep-learning technique that represents text as a vector and is an example of semantic relation modelling [47]. The goal of Word2Vec is to learn how to map words to a dense vector space, where the vectors represent the semantic relationships between words. Word2Vec plays a crucial role in representing short texts as vectors of semantic features by identifying the contextual meanings of words and capturing their semantic relationships. The Word2Vec model uses the Skip-gram architecture as shown in equation (Eqs 8 and 9), which predicts a word based on its context, thereby embedding words that appear in similar contexts close together in the embedding space [48].

$$\begin{array}{c} \frac{1}{T}\sum_{t=1}^{T}\sum_{-c\leq j\leq c,j\neq 0}\text{log}p(w_{t+j}|w_{t}) \end{array}$$ (8)

Where the *w*_*t*+*j*_ are the context words around *w*_*t*_ and *p*(*w*_*t*+*j*_|*w*_*t*_) are computed by the following equation (Eq 9).

$$\begin{array}{c} p(w_{t+j}|w_{t})=\frac{\text{exp}(v_{w_{t+j}}Tv_{w_{t}})}{\sum_{w=1}^{W}\text{exp}(v_{w}Tv_{w_{t}})} \end{array}$$ (9)

Here, $v_{w_{t}}$ is the ‘input’ vector representation of the target word, and $vv_{w_{t+j}}^{‘}$ are the ‘output’ vector representations of the context words.

For contextual semantic property (‘**Contextual Word Embedding**’), two types of ‘view’ are selected: (i) Bidirectional Encoder Representations from Transformers (BERT), and (ii) Generative Pre-trained Transformer (GPT). BERT is a pre-trained deep learning model that may be used to create vector representations for short texts. BERT is a cutting-edge method that generates high-quality contextual embeddings for text by combining deep bidirectional neural networks and transformer models [49]. This model can capture complicated and nuanced word relationships. The resulting vector form captures the semantic features of the entire text, allowing for a variety of downstream natural language processing (NLP) tasks (see Eq 10). BERT determines words’ contextual meaning by considering their surrounding context. This model can handle language nuances, including word ambiguity, and produce complex and informative representations of texts. BERT-based representations are highly effective for numerous NLP tasks, such as clustering, due to their ability to encapsulate semantic relationships.

$$\begin{array}{c} \text{max}\;\prod_{i=1}^{N}\;P(w_{i}|\text{context}) \end{array}$$ (10)

Where *w*_*i*_ represents the *i*th word in a sentence, and *N* is the total number of words.

As for GPT, it is a language model that uses deep learning to produce text vector representations [50]. In contrast with BERT, which generates vector representations based on a given text, GPT creates new text by predicting the next word in a sequence. However, the GPT-generated concealed representations can be used as vector representations for texts. Each new word is generated based on the hidden representations of the preceding words, enabling GPT to capture contextual relationships between words. GPT captures contextual relationships between words by predicting the following word depending on the words that came before it. This model enables it to comprehensively capture the meaning and coherence of texts, including syntactic structure, grammar, and semantic word relationships.

For semantic property (‘**Latent Semantics**’), we have used Latent Semantic Analysis (LSA) to analyze the relationships between a set of documents and the terms they contain by producing a set of concepts related to both the documents and terms [51]. It employs singular value decomposition (SVD) on the term-document matrix to reduce dimensionality, thereby capturing the latent semantic structures as shown in the following equation (Eq 11).

$$\begin{array}{c} A_{k}=U_{k}\Sigma_{k}V_{k}^{T} \end{array}$$ (11)

Where *U*_*k*_(*m* × *k*) contains the first *k* left singular vectors, Σ_*k*_(*k* × *k*) is a diagonal matrix with the top *k* singular values, and $V_{k}^{T}\left(k\times n\right)$ contains the first *k* right singular vectors; here, *m* represents the number of terms, and *n* represents the number of documents.

Based on all the selected single-view representations, the target short text will be transformed into vectors, and the vector representation will be further processed for reduction.

### Step 2: Vectors reduction based on PCA

In MVR, particularly focusing on STC, it is crucial to address the challenge of vector uniformity. The vectors derived from each view must be of equal dimensions to facilitate effective integration and analysis [52]. This uniformity is paramount to mitigating overfitting in the clustering method. PCA is used in this study to help achieve this consistency. PCA is a statistical procedure that uses an orthogonal transformation to convert a set of potentially correlated variables into a set of linearly uncorrelated variables known as principal components. The eigenvectors of the data’s covariance matrix define this transformation. This ensures that the first principal component has the most variance possible and that each component after that has the most variance possible, as long as it is orthogonal to the ones before [53]. The primary function of PCA in this context is to reduce the dimensionality of the data while maintaining as much of the data’s variability as possible.

This vector uniformity is significant for vectors from different views because it ensures that all vectors are of the same length, facilitating their combination. Dimensionality reduction via PCA reduces the data’s complexity and preserves its variability, making finding similarities between data points easier [54]. This enhanced uniformity improves the clustering performance and subsequent analytical methods by providing a consistent basis for comparison. The use of PCA in multi-view learning contexts has been shown to effectively handle the heterogeneity of different data views while maintaining important information [55]. The preservation of data variability during this process is crucial for maintaining the discriminative power of the features in subsequent analyses [56]. By ensuring that the vectors derived from BERT, GPT, TF-IDF, FastText, and GloVe are of consistent dimensions, we mitigate the risk of overfitting and enhance the overall robustness of the clustering process.

The mathematical representation of PCA involves the decomposition of the data covariance matrix. Consider *X* the data matrix, with each row representing a different observation and each column representing an additional variable. PCA transforms *X* into a new set of coordinates, the principal components, through the equation *Y* = *PX*. Here, *P* represents the matrix of eigenvectors of the covariance matrix of *X*, and *Y* is the main component. This transformation is significant for vectors from different views of the same length. As a result, dimensionality reduction makes it easier to combine different view representations by ensuring that vector dimensions are consistent. This makes the data more accessible to find similarities between them and improves the clustering and subsequent analytical methods, as listed below (see algorithm 1).

**Algorithm 1** Principal Component Analysis for Multi-View Representation

1: **function** PCA MVR (*X*[1], *X*[2], …, *X*[*m*], *d*)

2:  **for** each view *i* from 1 to *m* do

3:   Standardize $X\left[i\right]:X\left[i\right]\leftarrow \frac{X[i]-\mu [i]}{\sigma [i]}$

4:   Compute the sample covariance matrix:

5:    $\Sigma \left[i\right]\leftarrow \frac{1}{n-1}\sum_{k=1}^{n}(x_{k}\left[i\right]-\mu \left[i\right]){\left(x_{k}\left[i\right]-\mu \left[i\right]\right)}^{T}$

6:   Compute the eigenvalues λ[*i*] and eigenvectors *V*[*i*] of Σ[*i*]

7:   Define the transformation matrix *P*[*i*] ← [*v*_1_, *v*_2_, …, *v*_*d*_] with the *d* Eigenvectors associated with the *d* largest eigenvalues.

8:   Project the data *X*[*i*] into the PCA subspace:

9:    *Y*[*i*] ← *X*[*i*]*P*[*i*]

10:  **end for**

11:  Optionally, combine the reduced representations:

12:   *Y*_combined_ ← Combine views(*Y*[1], *Y*[2], …, *Y*[*m*])

13:  **return** *Y*_combined_

14: **end function**

### Step 3: Combining single views for MVR

**Fig 3:**
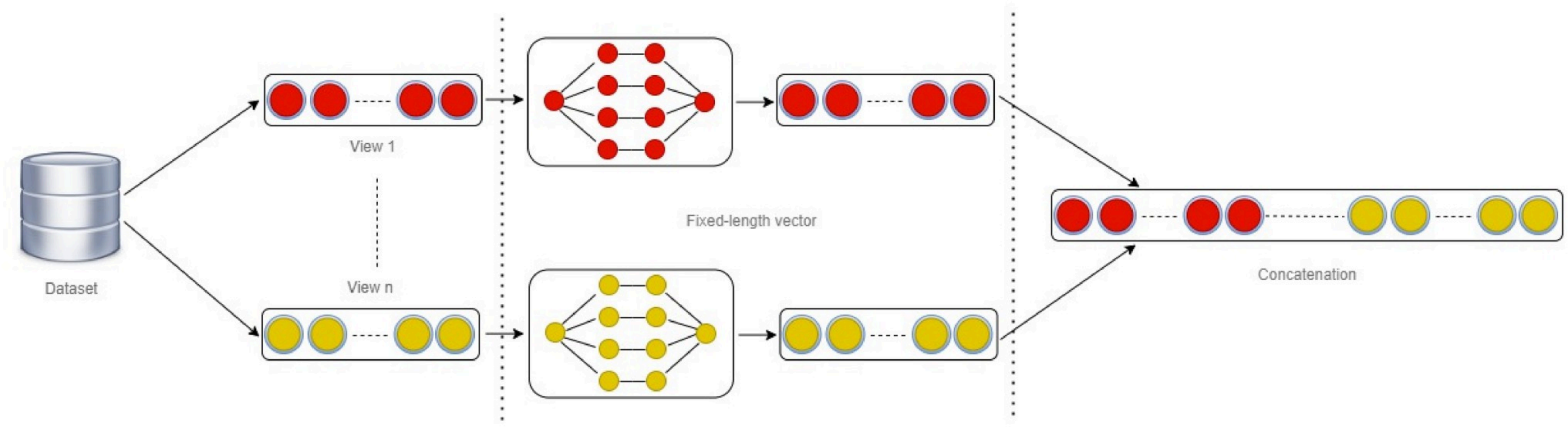
Overall framework for MVR.

The final step to designing MVR is to merge the single-views into MVR. The merging is done by a concatenating approach. The concatenation approach is chosen as the technique to merge single-views into MVR due to its simplicity and effectiveness in preserving the integrity of the original feature space. Integrating several perspectives from short text data while maintaining distinct characteristics from each standpoint to enhance the merged dataset is crucial. In other words, concatenation ensures no loss of information, unlike other methods that condense or abstract data. The strategy of employing a concatenation approach enhances clustering algorithms’ capacity to differentiate between fine data points by providing a wide range of features. The simplicity and interpretability of this approach are beneficial, as they eliminate the necessity for intricate conversions or substantial parameter modifications. For a clearer explanation, let *D* = {*d*_*i*1_, *d*_*i*2_, …, *d*_*iN*_} be the dataset of short text sequences, where *d*_*i*_ represents the *i*th data point in the dataset, and *N* is the total number of data points. Each data *d*_*i*_ is associated with a set of view representation denoted as $F_{i}=\left\{f_{ij_{1}},f_{ij_{2}},\ldots ,f_{ij_{m}}\right\}$, where *m* represents the total number of feature representations associated with *d*_*i*_, *j* represents the index for a specific view representation, and *f*_*ij*_ represents the *j* feature representation for *d*_*i*_. We adopt a concatenation approach to combine MVRs, as shown in Fig 3. We concatenate the feature vectors from all the view representations for each short text sequence *d*_*i*_. This approach results in a combined feature vector. The data representing view *x*_1_, view *x*_2_, and view *x*_*n*_ can be roughly characterised as follows (Eq 12):

$$\begin{array}{c} c_{i}=g(x_{1};f_{ij_{1}},f_{ij_{2}},\ldots ,f_{ij_{m}})\circ g(x_{2};f_{ij_{1}},f_{ij_{2}},\ldots ,f_{ij_{m}})\circ g(x_{n};f_{ij_{1}},f_{ij_{2}},\ldots ,f_{ij_{m}}), \end{array}$$ (12)

Where *g* denotes the encoding functions that map the original view data to the corresponding features according to *i* and *j* parameters. These characteristics are aggregated using the devised aggregation operator ∘ into a unified representation. The dimensionality of the combined feature vector will be the sum of the dimensionality of individual feature vectors from all the representations, *c*_*i*_.

At this stage, multiple feature sets from different views of text data are merged into a unified representation that captures the essence of each view to leverage the strengths and information from all the available feature sets. Using a PCA feature vector to fix the length ensures all feature vectors contribute equally without bias due to differing magnitudes or variances, as listed below (see algorithm 2).

**Algorithm 2** Combined MVR for STC

1: **Input**: Dataset of short text sequences {*D* = {*d*_*i*1_, *d*_*i*2_, …, *d*_*in*_}, where *d*_*i*_ is associated with multiple feature representations; $F_{i}=\left\{f_{ij_{1}},f_{ij_{2}},...,f_{ij_{m}}\right\}$

2: **Output**: Combined Multi-View Representation of short text sequences

3:  *MVR* ← Multi-View Representation/Short text

4: **Begin**

5:  Normalize the feature vectors to a fixed length using PCA

6:  **for** each feature vector *f*_*ij*_ in *F*_*i*_ **do**

7:   Apply PCA to reduce dimensionality

8:   $f_{ij_{\text{normalized}}}\leftarrow PCA\left(f_{ij}\right)$

9:   $F_{i_{\text{normalized}}}\leftarrow \;\text{Replace}\;\left(F_{i},f_{ij},f_{ij_{\text{normalized}}}\right)$

10:  **end for**

11:   Initialize Combined Vector

12:  **for** *j* = 1 to *m* **do**

13:   Combined Vector ← Concatenate (Combined Vector, *f*_*ij*_)

14:  **end for**

15:  Combined Feature Vectors ← Append (Combined Feature Vectors, ∘)

16:  **return** Combined Feature Vectors

17: **End**

## Implementation of MVR in STC

**Fig 4:**
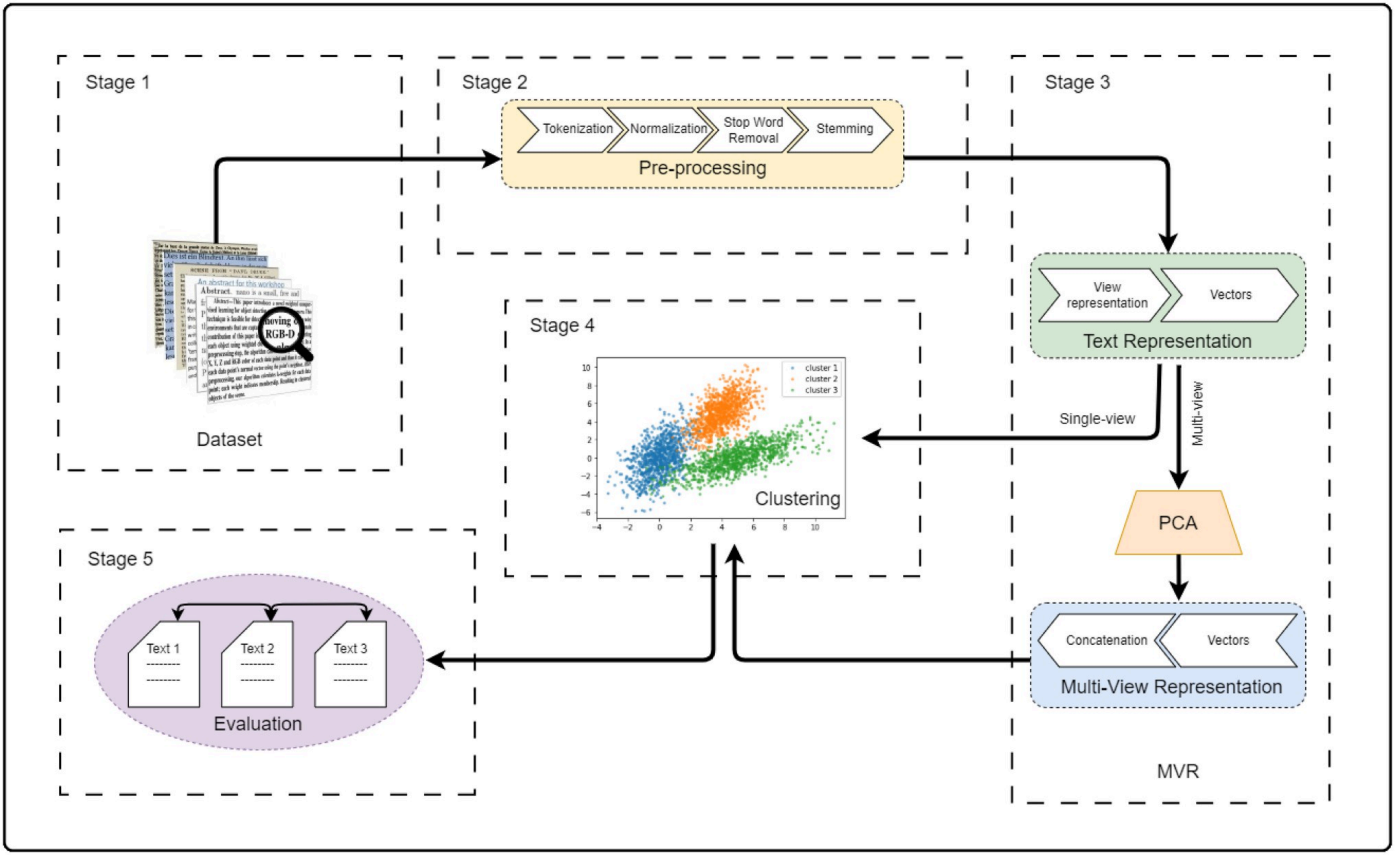
The overall framework of STC based on MVRs.

In order to find the optimal MVR for STC, we implement different types of MVR (a different combination of single-views) as the STC representation for a clustering algorithm (K-means). We consider the optimal MVR for STC as the STC model with the highest performance. The proposed study includes the following: Firstly, the study investigates the selection of optimal representation methods for integration in terms of effectiveness that are suitable for all types of STC. Secondly, a new strategy (see the MRV based on PCA section) for representing text based on MVR is presented to improve the performance of STC further. The objective is to determine the most practical combination of view representations for the short text model based on their applicability to different datasets and demonstrate how the proposed combined text representation improves the STC performance. Thus, the overall framework for finding the optimal MVR (see Fig 4) consists of five stages. These stages include (1) selecting a suitable short text dataset, (2) pre-processing dataset, (3) representing the text in different types of MVR, (4) performing clustering based on different types of MVRs and single-views and (5) evaluating the clustering models.

### Stage 1: Datasets

To implement MVRs in STC, we select three publicly available datasets, namely; *Google News*, *Twitter* and *StackOverflow* (see Table 1). These datasets are chosen because they have been extensively used in prior research to validate clustering methods [1, 13, 16, 29, 57]. Moreover, these datasets have been utilised as benchmarks for evaluating clustering techniques [16]. The selection of these datasets allows us to investigate and evaluate the performance of STC with MVR on various dataset types. Each dataset has unique characteristics that contribute to a broader understanding of text clustering. In contrast, the collection and analysis of these datasets comply with their respective sources’ terms and conditions. We ensured that using *Google News*, *Twitter* and *StackOverflow* adhered to their guidelines and policies for academic research purposes. All data were handled in a manner that respects the original content creators’ privacy and intellectual property rights. Below are the detailed descriptions of the datasets used in this study:

**Table 1:** Statistic information of the STC datasets.

Dataset	Number of texts	Average of words	Number of groups
Google News	11,109	6.23	152
Twitter	2,472	8.55	89
StackOverflow	16,407	5.13	20

The *Google News* dataset (http://news.google.com) is a valuable resource focuses on news article titles obtained from various online publishers. *Google News* provides a comprehensive collection of news article titles on various topics, including ‘politics’, ‘technology’, ‘sports’ and ‘entertainment’. This dataset contains the ‘titles’ and ‘excerpts’ of 11,109 news from *Google*, and every ‘title’ has an average of 6.23 words and grouped into 152 groups. The *Twitter* dataset (http://trec.nist.gov/data/tweets) is significant for informal social media short text documents widely used in social media analysis and IR research. The *Twitter* dataset concentrates on tweets obtained during the 2011 and 2012 microblog tracks of the *Text REtrieval* Conference initiative. This dataset provides an extensive database of real-time microblogging data, capturing disparate user-generated content across various topics and conversations. The *Twitter* dataset contains 2472 tweets, with an average of 8.55 words per tweet. These tweets are organised into 89 groups. The *StackOverflow* dataset (https://www.kaggle.com) is a dataset which a collection of question-and-answer from website for programmers and developers. The *StackOverflow* dataset contains user-generated programming-related questions, answers, comments and discussions. The raw dataset includes 16,407 questions titles, each containing an average of 5.13 words. These question titles are divided into 20 groups.

### Stage 2: Dataset pre-processing

Pre-processing of documents is important for a successful STC because unprocessed short text data cannot be directly clustered. Text data in short text documents may include, among other string types, words, sentences, numbers and symbols. The presence of noise in these strings can hinder grouping and information retravel (IR) [58, 59]. Many extraneous phrases in short texts can harm rather than enhance their representation; therefore, document pre-processing is essential in STC [60]. Accordingly, removing unnecessary words from a document is a fundamental pre-processing step [61]. [62] further indicated that even common English phrases, such as ‘go’, ‘gone’ and ‘going’, are generated via inflectional and derivational processes. Therefore, stemming helps consolidate the variant forms of a word into a single base form, increasing the density of relevant keywords in the text. Thus, the primary pre-processing processes in our study are tokenization, normalisation, stop word removal and stemming. We used the Natural Language Toolkit (NLTK), the comprehensive Python library that lists stop words for various languages and includes Porter stemmer; which is one of the most common and effective stemming tools that reduces words to their word stems.

### Stage 3: MVR as text representation

In this stage, we transform all the text in a dataset into vector representation with different views (representation), or the MVR. The MVR is a sophisticated approach in natural language processing. It amalgamates diverse data perspectives, incorporating semantic, syntactic, and lexical features to enrich text understanding. Therefore, in our approach, we combined different view representation destinations to capture the largest amount of information and overcome the short text’s lack of context. Furthermore, in our approach, we used PCA to unify the lengths of the different vector features and thus overcome the dominance of one representation at the expense of the other and unify them. The details of how the dataset documents are transformed into MVR have been explained in the MRV based on PCA section.

### Stage 4: Multi-view clustering short text

At this stage, the short text (dataset) will be clustered using the K-means algorithm, which will be used to show that the proposed MVR can enhance the performance of the clustering algorithm, and K-means has been extensively used for clustering tasks by previous researchers [29, 32, 57, 63]. K-means is a traditional clustering algorithm that aims to divide a given dataset into K, a predefined cluster. The algorithm iteratively assigns each data point to the nearest cluster centroid and updates the centroid based on the weighted average of the allotted data points. This procedure continues until convergence when the assignments and centroids no longer substantially vary. In this paper, the K-means cluster short text is based on both single-views and different types of MVR.

In this work, we used the K-means clustering method for STC based on MVR as explained in the following: Let $F^{\left(v\right)}\in R^{d_{v}*n}$ denote the features in the *v*th view with *n* vectors and *d* dimensional visual features, $FX^{\left(v\right)}inR^{d_{v}*k}$ be the centroid matrix for the *v*th view and $G^{\left(v\right)}\in R^{n*k}$ be the clustering indicator matrix for the *v*th view. Given the *M* types of different features, *v* = 1, 2, …, *M* The simplest method to utilise all feature views is to concatenate all features and execute the clustering algorithm. The main idea is to define *k* centroids, one for each cluster. The objective function *J* is given as follows (Eq 13):

$$\begin{array}{c} \text{min}\;J=\sum_{j=1}^{k}\sum_{i=1}^{n}{∥x_{i}^{\left(j\right)}-c_{j}∥}^{2} \end{array}$$ (13)

Where $∥x_{i}^{\left(j\right)}-c_{j}{∥}^{2}$ is a chosen distance measure between a data point $x_{i}^{\left(j\right)}$ and the cluster centre *c*_*j*_. At this stage, the K-means algorithm is employed to investigate the efficacy of the proposed MVR model for text representation in STC, providing a baseline for comparison with traditional text representation methods as listed below (see Algorithm 3).

**Algorithm 3** K-means Clustering Algorithm based on MVRs

1: **Input**: Combined Feature Vectors (Combined MVR for STC)

2: **Set** the number of clusters *k*

3: Select *k* instances from Combined Feature Vectors as the initial cluster centers.

4:  **while** centroids change **do**

5:   **for** each instance in Combined Feature Vectors **do**

6:    Assign the instance to the closest cluster centroid.

7:   **end for**

8:   Recalculate the cluster centroids.

9:  **end while**

10: **Output**: Clusters with their respective centroids.

11: **End**

The time complexity of the STC model for a short dataset can be divided into four categories: preprocessing, representation, fixed length using PCA, and clustering. The time complexity for preprocessing is *O*(*n*), where *n* denotes the number of tokens. The time complexity for BERT/GPT encoding is *O*(*n*^2^.*d*_*b*_) where *n* is the sequence length and *d*_*b*_ is the model dimension. For FastText/GloVe embedding lookup, it is *O*(*n*.*d*_*w*_), where *n* is the number of tokens and *d*_*w*_ is the word vector dimension. For TF-IDF calculation, it is *O*(*n*.*m*), where *m* denotes the vocabulary terms. The time complexity of the PCA algorithm is expressed as *O*(*N*.*D*^2^ + *D*^3^), where *N* is the number of samples and *D* indicates the input dimensionality. The time complexity of the clustering stage using the K-means algorithm is *O*(*N*.*k*.*t*.*d*_*pca*_), where *k* represents the number of clusters, *t* represents the number of iterations, and *d*_*pca*_ represents the reduced dimensionality after PCA. The PCA and K-means methods are computationally intensive, emphasizing the importance of efficient implementation and the potential for parallelization to handle the computational workload effectively.

### Stage 5: Evaluation metrics

In this stage, performance metrics were utilised to evaluate the MVR by comparing the clusters generated using the MVRs and single-view representations by comparing the clusters generated by the model to the correct clusters [64, 65]. Three common metrics for clustering performance measures were used: normalised mutual information (NMI) [66], Adjusted Mutual Information (AMI) [67] and purity (P) [68]. Where represents the total number of texts. We assume that *n*_*i*_ represents the number of short texts in the *i* correct topic and *n*_*j*_ denotes the number of short texts in the *j* inferred cluster. *n*_*ij*_ is the number of short texts that simultaneously appear in two clusters. *K* denotes the number of clusters, whereas max_*j*_(*n*_*ij*_) represents the maximum number of documents in every cluster. The fundamental evaluation matrices can be used to efficiently find the optimum evaluation. Purity, NMI and AMI can be formally expressed as follows (Eqs 14–16):

$$\begin{array}{c} \text{NMI}=\frac{\sum_{ij}\;n_{ij}\text{log}\frac{n.n_{ij}}{n_{i}n_{j}}}{\sqrt{\left(\sum_{i}n_{i}\text{log}\frac{n_{i}}{n})(\sum_{j}\;n_{j}\;\text{log}\frac{n_{j}}{n}\right)}}, \end{array}$$ (14)

AMI=∑ij(nij2)-(∑i(ni2)∑j(nj2))/(n2)12(∑i(ni2)∑j(nj2))-(∑i(ni2)∑j(nj2))/(n2), (15)

$$\begin{array}{c} \text{Purity}\left(P\right)=\frac{1}{n}\sum_{k}\underset{j}{\text{max}}(n_{ij}) \end{array}$$ (16)

The higher purity, NMI and AMI scores indicate superior clustering quality. When a perfect match is attained in cluster assignments across the entire corpus, they are equal to one. NMI and AMI penalise texts unnecessarily divided from a single true cluster into multiple inferred clusters. AMI is a more stringent metric because it penalises the objectionable merging of texts from distinct correct clusters into the same inferred cluster.

## Evaluation and discussion

In this study, we present the results of implementing the proposed MVRs on STC across three datasets. As mentioned in the MRV based on PCA section, we selected eight single-view representation types: BERT, GPT, FastText, Word2Vec, LSA, GloVe, TF-IDF, and BOW. These single-view types were then combined to form MVRs. In K-means clustering, cosine similarity was used as the internal similarity metric with 50 random initial centroids. To determine the best MVR for STC, we applied all types of single-views and their combinations.

### Experimental results

The experimental setup to identify the best combination of single-views for MVR involves applying each single-view and every combination of single-views across the three previously mentioned datasets. Subsequently, Tables 2 to 4 depict the best results of STC for each type of combination of single-view (MVR) and single-view.

**Table 2:** Performance of STC with the best result for single-view and each type of MVRs on the *Twitter* dataset.

Single-view/MVR	Metric		
	Purity	AMI	NMI
Single-view	0.7932848	0.731368	0.7872638
MVR (2-views)	0.8114887	0.7368681	0.7920219
MVR (3-views)	0.8329935	0.7336794	0.8058143
MVR (4-views)	0.8547249	0.7548567	0.8243974
MVR (5-views)	**0.8900485**	**0.8053227**	**0.8678961**
MVR (6-views)	0.8238497	0.7199985	0.781627
MVR (7-views)	0.7633495	0.6694823	0.7412583
MVR (8-views)	0.7536408	0.6542413	0.7291702

**Table 3:** Performance of STC with the best result for single-view and each type of MVRs on the *GoogleNews* dataset.

Single-view/MVR	Metric		
	Purity	AMI	NMI
Single-view	0.7450072	0.737034	0.7785684
MVR (2-views)	0.7597371	0.7412431	0.7816651
MVR (3-views)	0.7786496	0.7535376	0.7959442
MVR (4-views)	0.8008102	0.7830479	0.8256357
MVR (5-views)	**0.8643356**	**0.844621**	**0.8900074**
MVR (6-views)	0.7120382	0.7018642	0.7529024
MVR (7-views)	0.6754807	0.6684533	0.7355162
MVR (8-views)	0.6598383	0.65452	0.7121945

**Table 4:** Performance of STC with the best result for single-view and each type of MVRs on the *StackOverflow* dataset.

Single-view/MVR	Metric		
	Purity	AMI	NMI
Single-view	0.6034083	0.542754	0.5649979
MVR (2-views)	0.6417992	0.5892176	0.6006254
MVR (3-views)	0.6739794	0.5917716	0.613781
MVR (4-views)	0.7048936	0.6333439	0.6553489
MVR (5-views)	**0.7754422**	**0.6934234**	**0.7154282**
MVR (6-views)	0.6376973	0.554917	0.5669152
MVR (7-views)	0.5976973	0.524642	0.5566414
MVR (8-views)	0.5815457	0.5215416	0.5435913

In Tables 2–4, the second column represents the best results for each type of MVR or single-view across the three datasets. As shown in these tables, BERT has achieved the highest results among single-views, outperforming the other seven types (GPT, FastText, Word2Vec, LSA, GloVe, TF-IDF, and BOW). Consistently, the 5-views MVR—a combination of BERT, GPT, TF-IDF, FastText, and GloVe—has yielded the highest results among all MVRs and single-views. Based on the results from Tables 2–4, we conclude that the optimal MVR for STC (given the standard datasets of Twitter, Google News, and Stack Overflow) is the 5-views combination of BERT, GPT, TF-IDF, FastText, and GloVe.

However, the performance of STC significantly increased when these five views were combined. The combined views achieved the highest scores with the *Twitter* dataset, resulting in a Purity of 89.00%, an AMI of 80.53%, and an NMI of 86.79%, as shown in Table 2. Similarly, the best performance for the Google News dataset, as shown in Table 3, was a Purity of 86.43%, an AMI of 84.46, and an NMI of 89.00%. Finally, the best performance for the Stack Overflow dataset with the five-view representation, as shown in Table 4, was a Purity of 77.54%, an AMI of 69.43, and an NMI of 71.54%.

The theoretical analysis indicates that the BERT-based single-view outperforms other single-views due to the superior knowledge learned through its deep contextual understanding. As a transformer-based model that can capture context in text more effectively, it is crucial in STC, where context is still unclear due to the brevity of texts [49]. Additionally, the remarkable results achieved by the 5-views MVR comprising BERT, GPT, GloVe, FastText, and TF-IDF underscore the theoretical advantage of combined methods in machine learning. Therefore, combining multi-views leads to diverse linguistic and semantic features captured differently by each model. The BERT and GPT give embeddings that capture deep contextual information, while FastText and GloVe are more resilient to fluctuations in word morphology. On the other hand, TF-IDF focuses on word frequency metrics, quantifying the occurrence of a word in a specific document relative to its frequency across other documents. This approach effectively highlights rare but important terms, which certain embedding models may overlook. Thus, integrating these diverse views leads to a more comprehensive, more prosperous and more discriminative set of features feature space, enhancing the clustering algorithm’s ability to discern more nuanced patterns and relationships within the data. These results underscore the importance of combining different single-view representations to leverage their unique strengths.

**Fig 5:**
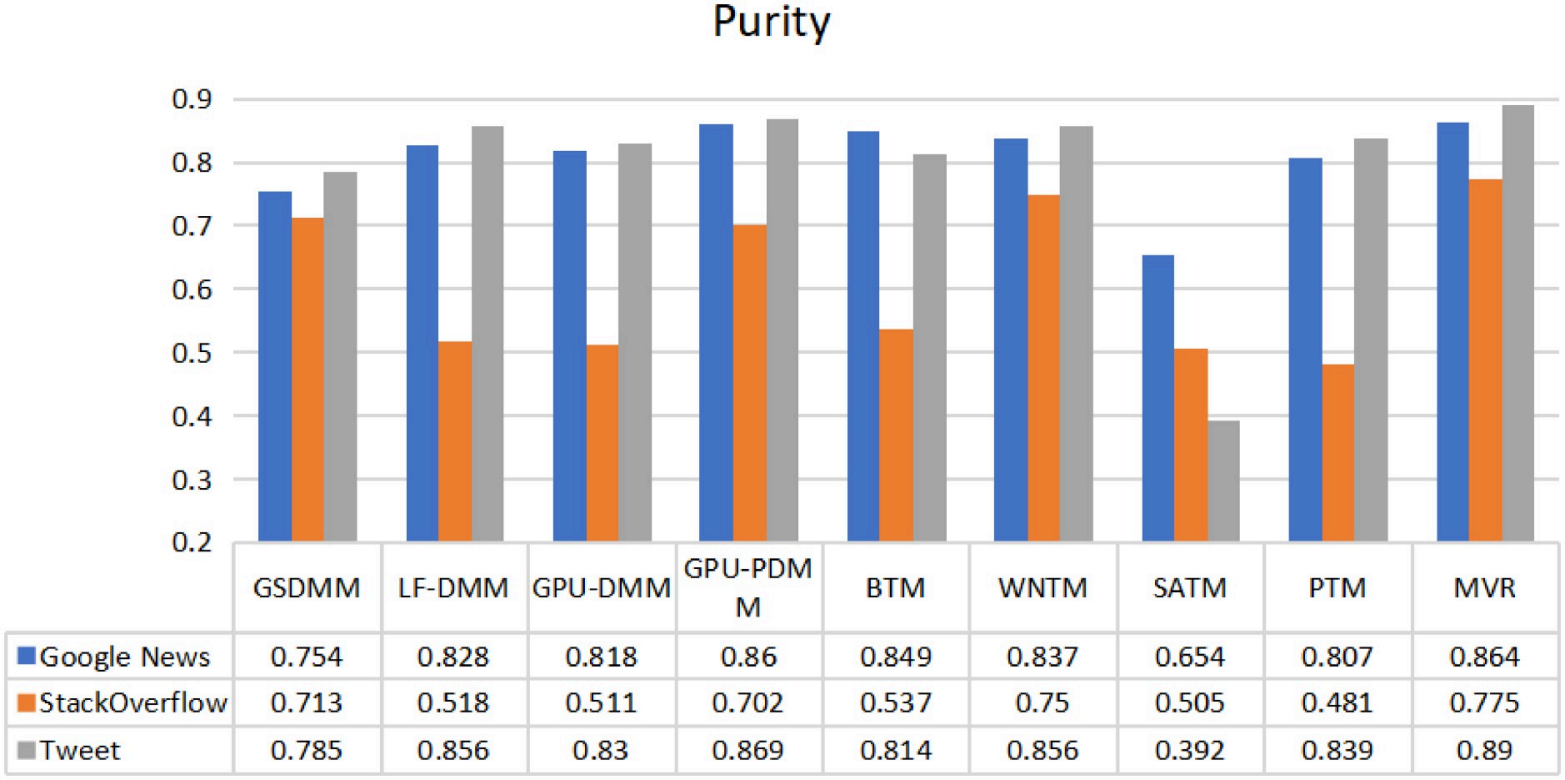
2-dimensional visualization of K-means clustering for the Twitter dataset.

**Fig 6:**
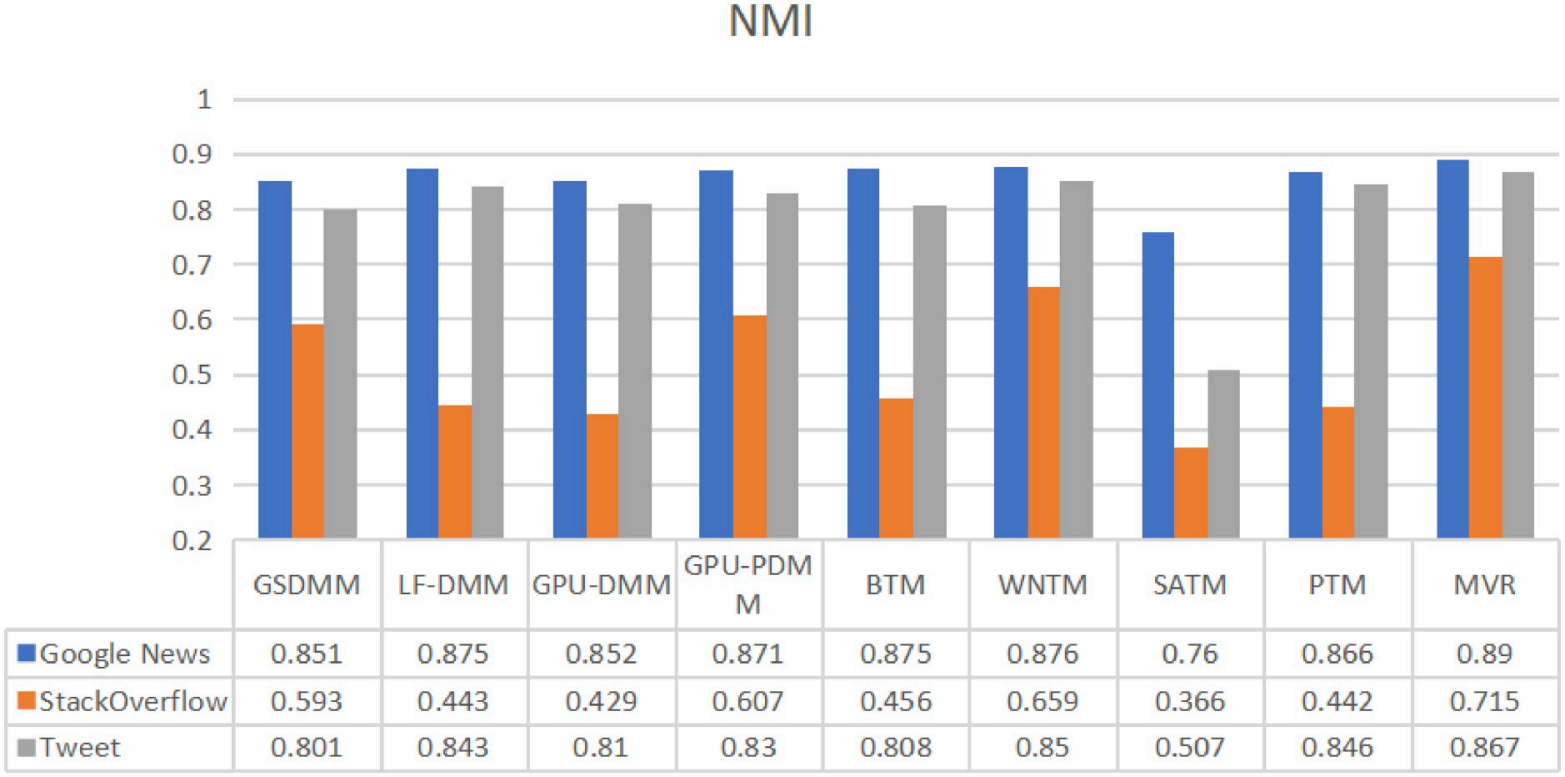
2-dimensional visualization of K-means clustering for the Google News dataset.

**Fig 7:**
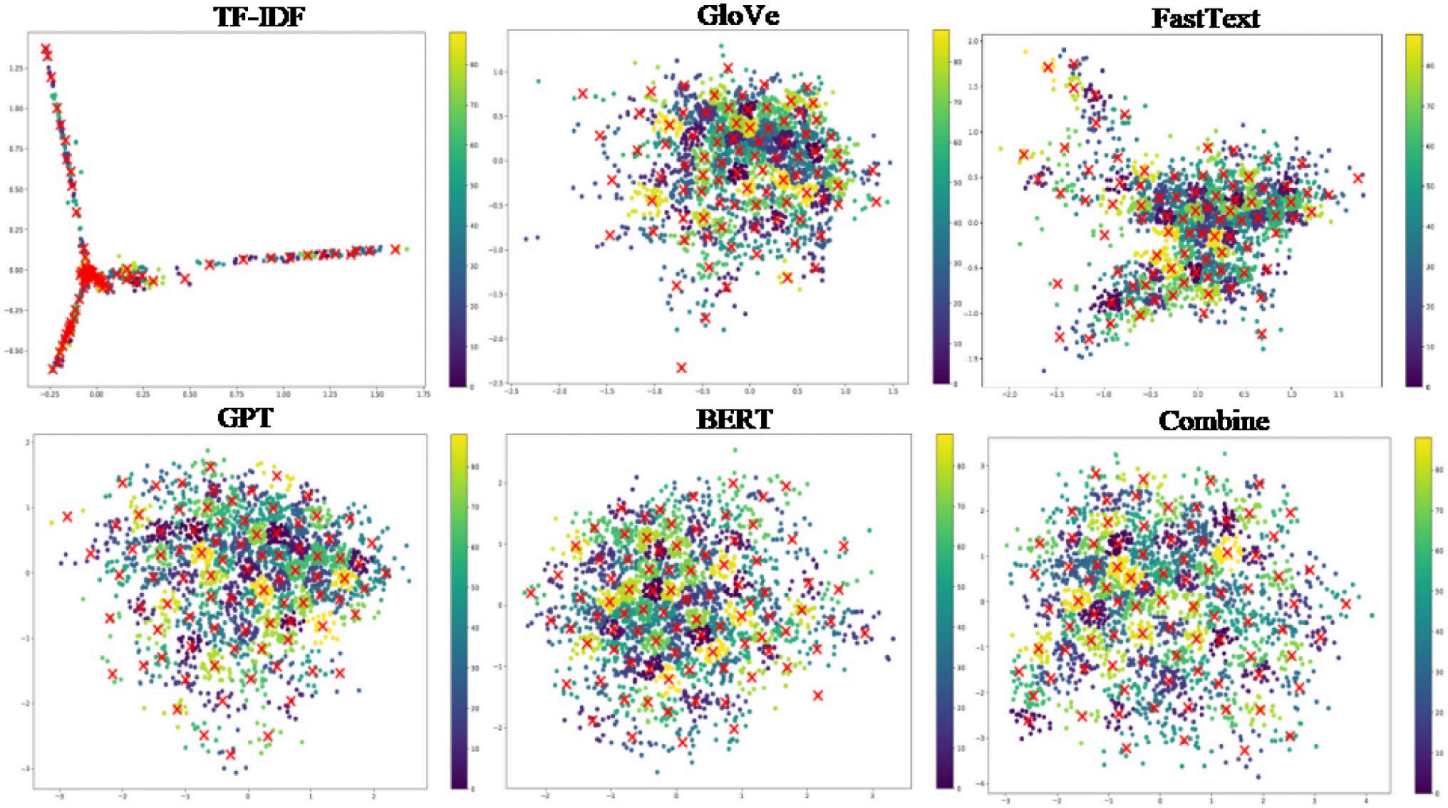
2-dimensional visualization of K-means clustering for the Stack Overflow dataset.

Figs 5–7 depict 2-dimensional visualizations of K-means clustering for the Twitter, Google News, and Stack Overflow datasets, respectively. Each figure shows the clustering results, with different colours representing various clusters. These subplots show stable cluster assignments with clear edges and center points for data point assignments for various view types. These visualizations highlight how K-means effectively groups similar data points based on feature representations, showing the refinement and convergence of clusters over combined representations.

## Discussion

In this work, we observe that combining all types of views does not necessarily produce the best MVR to enhance STC. Our work demonstrates the exceptional effectiveness of the 5-view combination, due to its ability to incorporate a well-rounded blend of features from different representation methods. This synergy allows the STC algorithm to identify more complex patterns and relationships in the data, resulting in enhanced clustering results.

We experimented with single-view and various types of MVR to find the optimal STC for each dataset type. The results of the experiments detailed in Tables 2–4 show that our method significantly improves the K-means clustering algorithm results. In Table 2, we experimented with different text representation methods based on the number of combined views using the Twitter dataset. BERT achieved the highest result for a single view with a score of 0.79. The combination of BERT, GPT, TF-IDF, FastText, and GloVe, which integrates five view methods, achieved the highest result with a score of 0.89, the highest for our proposed method. Likewise, Table 3 presents the experimental results obtained using the GoogleNews dataset, wherein various text representation methods were evaluated based on the number of combined views. Among the single views, BERT achieved the highest result, with a score of 0.74. In the proposed method, the configuration BERT, GPT, TF-IDF, FastText, and GloVe, which incorporated five views, yielded the highest result with a score of 0.86. This score represents the highest value achieved for the proposed method.

Similarly, Table 4 shows that the BERT feature representation produced the highest value compared with the single view, which scored 0.60. When the combination was extended to five views, BERT, GPT, TF-IDF, FastText, and GloVe exhibited the highest result for a single view with a score of 0.77 compared with the various views, indicating that the results are improved using the proposed method. The findings highlight the effectiveness of MVA for STC. The results indicate that efficacy initially improves as the number of combined views increases to five. The selected five views provide a balanced and optimal combination for the Twitter dataset, capturing various linguistic, semantic, and contextual aspects of short text data, which enables the clustering algorithm to identify significant patterns. These views collectively provide the necessary information for effectively clustering the Twitter dataset. However, adding more than five views yields suboptimal results, as the MVR approach has limitations, including declining performance due to increased noise, duplication, or computational complexity.

**Fig 8:**
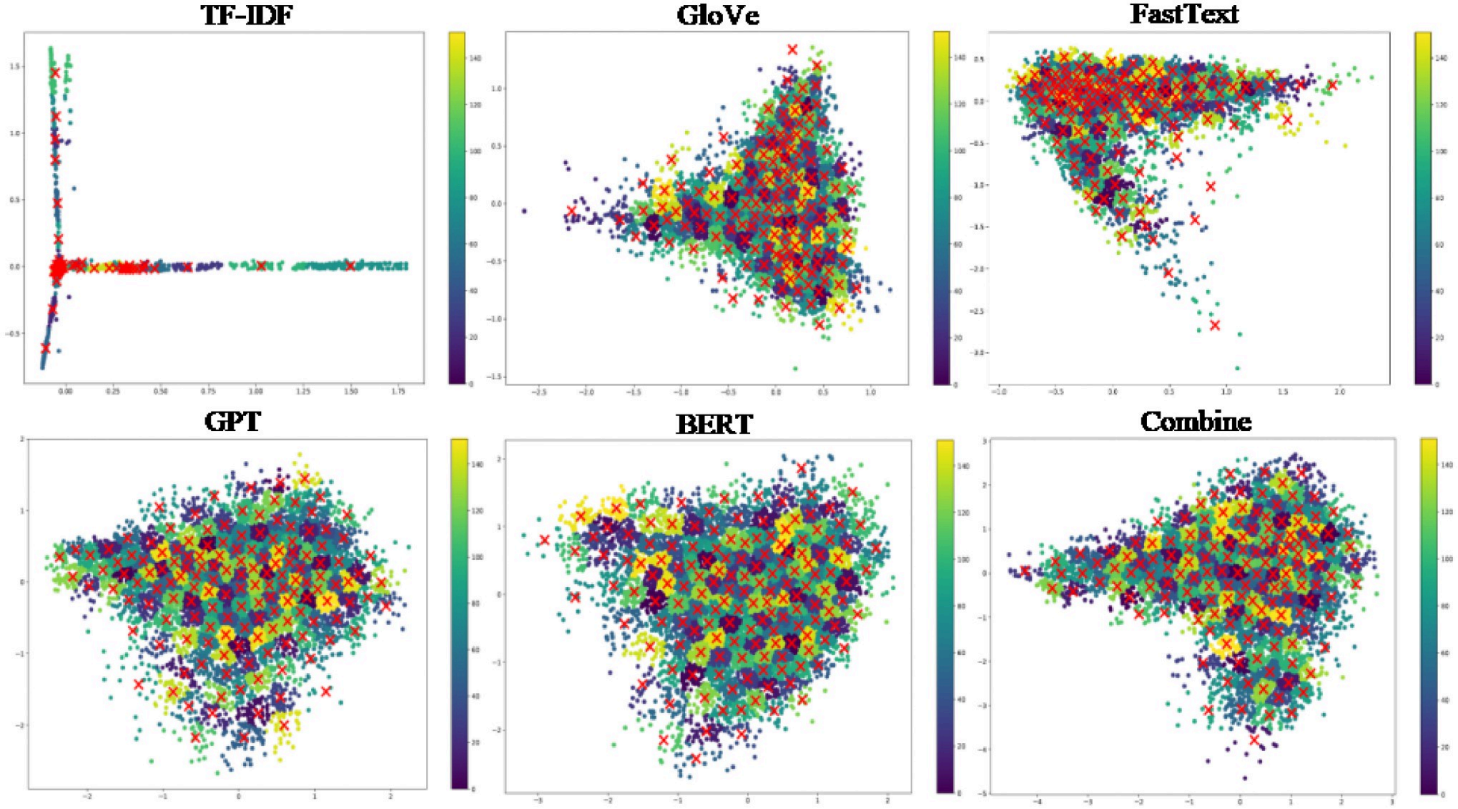
Comparison of the performance of the proposed method with other methods for three datasets using a purity metric.

**Fig 9:**
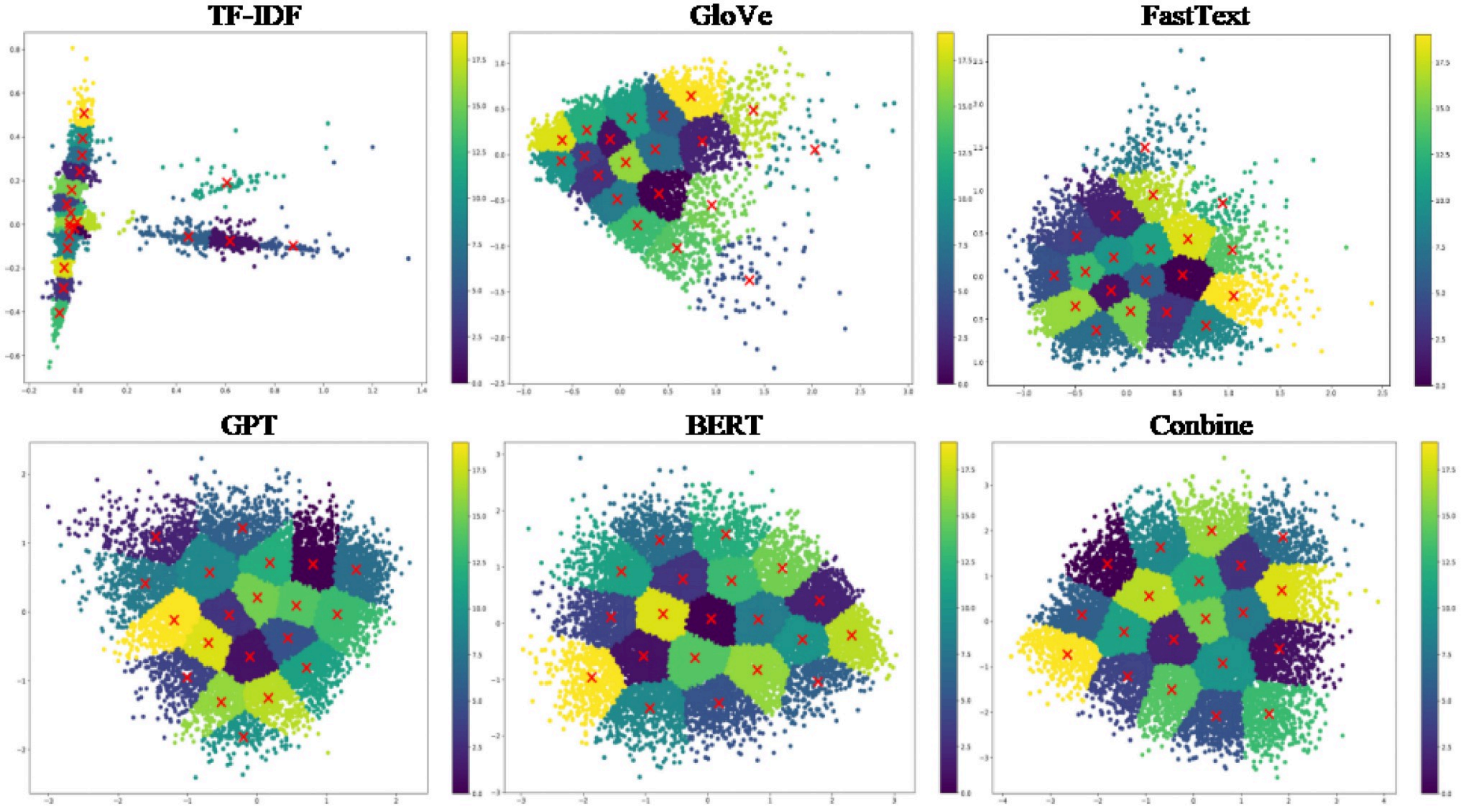
Comparison of the performance of the proposed method with other methods for three datasets using a NMI metric.

In Figs 8 and 9, The same datasets (Google News, Stack Overflow, and Twitter) were used by [16] to review various STC modeling techniques based on three categories: Dirichlet multinomial mixture, global word co-occurrences, and self-aggregation. All the models utilized a single-view representation, and the STC models were implemented in their open-source library named STTAM. By comparing our MVR-enhanced STC against all the surveyed STC models by [16], we can gain a general view of how our proposed optimal MVR in STC performs in comparison to different STC models that employ single-views from other types of STC. The comparison demonstrates that our MVR approach not only captures a richer array of textual features but also enhances clustering outcomes by offering a more nuanced understanding of text groupings.

Furthermore, we conducted a statistical test to determine if our proposed MVR method is superior to other methods, as shown in Figs 8 and 9, using purity and NMI as evaluation metrics. The selected statistical test included a paired t-test. Table 5 presents the results of these tests for the MVR model using both metrics—purity and NMI—across three datasets: Google News, StackOverflow, and Twitter. The results across both metrics and datasets indicate that the MVR model significantly outperforms the comparison groups or baselines. The t-tests demonstrate that the MVR model performs better in all cases; both Purity and NMI metrics show statistically significant differences compared to other models, with t-statistic values and p-values below 0.05. This signifies superior performance to the MVR model’s effectiveness for various data types, making it a robust choice for diverse applications.

**Table 5:** MVR performance t-test result.

Dataset	Purity Metrix (t-test)		NMI Metrix (t-test)	
	t-statistic	p-value	t-statistic	p-value
Google News	2.6426	0.0332	2.6656	0.0322
StackOverflow	4.7136	0.0021	5.8179	0.0006
Tweet	4.9796	0.0025	5.2231	0.0019

## Conclusion and future work

This study addresses the challenges in STC due to the failure to find suitable representation methods to capture similarities between texts. The aim of this study was achieved by designing a new multi-view approach applied to STC. Our proposed method was applied to three datasets common in the literature (Twitter, Google News, and StackOverflow) and proved competitive with state-of-the-art methods. Unlike the previously proposed methods, our algorithm relies on extracting features using different representations and combining multi-views, thus giving it a solid theoretical background for optimal multi-view combination strategies. Firstly, we constructed Multi-View Representations (MVRs) based on different single-view sets using fixed-length concatenation facilitated by Principal Component Analysis (PCA). Secondly, we determined the most suitable MVR for STC based on the performance evaluations across various MVR sets. Despite its simplicity, our experiments demonstrated the effectiveness of our MVRs with different datasets. In our future work, we plan to couple our proposed method with a feature reduction technique to integrate a combining step followed by a reduction step. This approach aims to enhance STC by extracting more extensive and comprehensive information. Additional future work could explore applications for merging multi-view clustering partitions in fields beyond text mining and natural language processing. This extension would test the robustness and applicability of our approach in other complex domains.
